# 骨髓增生异常肿瘤疾病进展和白血病转化过程中基因突变动态变化研究

**DOI:** 10.3760/cma.j.cn121090-20240708-00254

**Published:** 2025-03

**Authors:** 欣 严, 海洋 陈, 莲 王, 雨露 田, 岩 顾, 娜 刘, 峥 葛

**Affiliations:** 1 东南大学附属中大医院血液科，东南大学血液病研究所，南京 210009 Department of Hematology, Zhongda Hospital, School of Medicine, Southeast University, Institute of Hematology Southeast University, Nanjing 210009, China; 2 盱眙县人民医院，盱眙 211700 Xuyi People's Hospital, Xuyi 211700, China

**Keywords:** 骨髓增生异常肿瘤, 疾病进展, 白血病转化, 动态变化, Myelodysplastic neoplasms, Progressive disease, Leukemic transformation, Dynamic changes

## Abstract

**目的:**

分析骨髓增生异常肿瘤（Myelodysplastic neoplasms, MDS）疾病进展（Progressive disease, PD）/白血病转化（Leukemic transformattion, LT）组和非PD/LT组患者病程中基因突变动态变化差异，探索在MDS发生PD/LT过程中起关键作用的基因突变。

**方法:**

收集2019年5月至2023年8月于东南大学附属中大医院就诊的至少有２次高通量二代测序（Next generation sequencing, NGS）基因突变结果的84例MDS患者，比较PD/LT组和非PD/LT组患者病程中基因突变动态变化差异。

**结果:**

①84例患者中男性51例，女性33例，初次测序时中位年龄69（31～95）岁。PD组20例，LT组13例，非PD/LT组51例。初次测序时PD/LT组中位骨髓原始细胞比例高于非PD/LT组（1.6％对0.4％，*P*＝0.013）。②84例患者初次测序时基因突变检出率较高的依次为ASXL1（21例，25.0％）、TP53（17例，20.2％）、TET2（12例，14.3％）、DNMT3A（11例，13.1％）、U2AF1（11例，13.1％）；PD/LT组患者初次测序时中位基因突变个数高于非PD/LT组（2个对1个，*P*＝0.014）；PD/LT组初次测序时TET2（27.3％对5.9％，*P*＝0.010）、SETBP1（15.2％对2.0％，*P*＝0.033）、RUNX1（18.2％对2.0％，*P*＝0.013）突变比例高于非PD/LT组。③84例患者病程中检出率较高的新增突变（Ⅰ类突变）/克隆扩增突变（Ⅱ类突变）依次为TP53（9例，10.7％）、TET2（7例，8.3％）、ASXL1（7例，8.3％）、RAS旁路突变（7例，8.3％）；PD/LT组中位Ⅰ/Ⅱ类基因突变数目显著高于非PD/LT组（2个对0个，*P*<0.001）。PD/LT组患者Ⅰ/Ⅱ类RAS旁路（21.2％对0，*P*＝0.001）、TP53（27.3％对0，*P*<0.001）、TET2（18.2％对2.0％，*P*＝0.013）突变比例显著高于非PD/LT组。④PD/LT组75.0％（9/12）患者TP53突变为Ⅰ/Ⅱ类突变；非PD/LT组患者TP53突变皆为克隆缩小（Ⅲ类突变）（5/8，62.5％）或克隆稳定突变（Ⅳ类突变）（3/8，37.5％）。PD/LT组87.5％（7/8）的患者RAS旁路突变为Ⅰ/Ⅱ类突变；非PD/LT组患者仅有1例病程中有RAS旁路突变，为Ⅳ类突变。

**结论:**

PD/LT组患者初次测序时中位骨髓原始细胞比例和基因突变数目高于非PD/LT组；TET2、SETBP1、RUNX1突变比例高于非PD/LT组。PD/LT组中位Ⅰ/Ⅱ类基因突变数目和Ⅰ/Ⅱ类TP53、RAS旁路、TET2基因突变比例高于非PD/LT组。Ⅰ/Ⅱ类TP53和RAS旁路突变可能在MDS发生PD/LT过程中起关键作用。

## Abstract (en)

**Objective:**

To investigate the key genetic mutations during the progressive disease（PD）/leukemic transformation（LT）course in MDS by analyzing the dynamic changes of genetic mutations in patients with myelodysplastic neoplasms（MDS）with or without PD/LT.

**Methods:**

This study enrolled 84 patients with sequential MDS from May 2019 to August 2023 at ZhongDa Hospital Southeast University and used the next generation sequencing to detect gene mutations. The dynamic changes of genetic mutations in patients with MDS with or without PD/LT were retrospectively analyzed.

**Results:**

①This study analyzed data from 84 patients diagnosed with MDS with a median age of 63（range: 31–95）years and consisting of 51 males and 33 females. Participants were distributed to the PD cohort（*n*＝20）, LT cohort（*n*＝13）, and non-PD/LT cohort（*n*＝51）. Patients from the PD/LT cohorts demonstrated a higher proportion of bone marrow blasts than the non-PD/LT cohort at the first sequencing（1.6％ *vs*. 0.4％, *P*＝0.013）. ②The most frequently mutated genes that were detected at first sequencing were ASXL1（*n*＝21, 25.0％）, TP53（*n*＝17, 20.2％）, TET2（*n*＝12, 14.3％）, DNMT3A（*n*＝11, 13.1％）, and U2AF1（*n*＝11, 13.1％）. Further, patients from the PD/LT cohorts exhibited a higher median number of mutated genes than the non-PD/LT cohort（2 *vs*.1, *P*＝0.014）at first sequencing. TET2（27.3％ *vs*. 5.9％, *P*＝0.010）, SETBP1（15.2％ *vs*.2.0％, *P*＝0.033）, and RUNX1（18.2％ *vs*. 2.0％, *P*＝0.013）mutations were enriched in the PD/LT cohorts than in the non-PD/LT cohort. ③The most frequently detected acquired mutations（Ⅰ mutations）and clonally expanded mutations（Ⅱ mutations）were TP53（*n*＝9, 10.7％）, TET2（*n*＝7, 8.3％）, ASXL1（*n*＝7, 8.3％）, and RAS pathway（*n*＝7, 8.3％）. Furthermore, patients from the PD/LT cohorts showed a higher median number of Ⅰ/Ⅱ genes than the non-PD/LT cohort（2 *vs*.0, *P*<0.001）, and Ⅰ/Ⅱ RAS pathway（21.2％ *vs*. 0, *P*＝0.001）, TP53（27.3％ *vs*. 0, *P*<0.001）, and TET2（18.2％ *vs*. 2.0％, *P*＝0.013）mutations were enriched in PD/LT cohorts than in the non-PD/LT cohorts. ④Most of the TP53 mutations（9/12, 75.0％）in PD/LT cohorts were Ⅰ/Ⅱ mutations, whereas all of the TP53 mutations in non-PD/LT cohort were clone-decrease mutations（Ⅲ mutations）（5/8, 62.5％）or clone-stable mutations（Ⅳ mutations）（3/8, 37.5％）. Most of the RAS pathway mutations（7/8,87.5％）in the PD/LT cohorts were Ⅰ/Ⅱ mutations, whereas only one patient in the non-PD/LT cohort demonstrated RAS pathway mutations, which belonged to Ⅳ mutations.

**Conclusion:**

Patients from the PD/LT cohorts demonstrated a higher proportion of bone marrow blasts and a higher median number of mutations than the non-PD/LT cohort at first sequencing; TET2, SETBP1, and RUNX1 mutations were enriched in the PD/LT cohorts than in the non-PD/LT cohort at first sequencing. Patients from the PD/LT cohorts exhibited a higher number of Ⅰ/Ⅱ mutations than the non-PD/LT cohort. Further, Ⅰ/Ⅱ TP53, RAS pathway, and TET2 mutations were enriched in the PD/LT cohorts, and Ⅰ/Ⅱ TP53 and RAS pathway mutations may contribute to the PD/LT.

骨髓增生异常综合征（Myelodysplastic syndromes, MDS）是一种克隆性造血干细胞恶性疾病，其特征是血细胞减少、髓系细胞一系或多系发育异常、无效造血及演变为急性髓系白血病（Acute myeloid leukemia, AML）风险增高[1]。2022年WHO更新的髓系肿瘤分类中，将其新命名为骨髓增生异常肿瘤（Myelodysplastic neoplasms, MDS）以更准确描述此类疾病的肿瘤属性[2]。MDS患者在病程中会发生疾病进展（Progressive disease, PD），出现原始细胞增高、贫血加重、中性粒细胞和血小板减少等表现[3]，约30％的MDS患者最终会进展为AML[4]。既往研究表明，骨髓原始细胞数目增高，修订版国际预后积分系统（Revised international prognostic scoring system, IPSS-R）细胞遗传学分组差或极差，DNMT3A、NPM1突变是MDS患者发生白血病转化（Leukemic transformation, LT）的独立危险因素[5]–[9]，基因突变在髓系肿瘤发生LT过程中起重要作用[10]–[11]。既往序贯测序研究显示，病程中新增的激酶信号通路和黏蛋白复合物通路相关基因突变是促使MDS患者发生LT的重要因素[12]–[13]。本研究通过对我院84例有序贯测序结果的MDS患者病程中基因突变进行动态变化分析，探索在MDS发生PD/LT过程中可能起关键作用的基因突变。

## 病例与方法

1. 病例：本研究为回顾性队列研究，将2019年5月20日至2023年8月16日于东南大学附属中大医院血液科就诊的依据WHO2022分型标准[3]诊断或重新诊断的有序贯测序结果的84例MDS患者纳入本研究，纳入患者至少有2次二代测序结果且每次测序时的临床指标与实验室检查结果完整，包括性别、年龄等一般资料，血细胞计数和分类计数、骨髓穿刺涂片细胞形态学检查、骨髓活检组织切片病理学检查、染色体核型分析检测结果。本研究通过东南大学附属中大医院伦理委员会批准后实施（批件号：2023ZDSYLL051-P01）。

2. 基因突变分析：取患者骨髓并分离单个核细胞，常规提取DNA且制备DNA全基因组文库。使用PCR引物扩增目的基因组（涵盖58个血液肿瘤相关基因），将目标区域DNA富集后，采用Illumina Hiseq测序平台进行测序。等位基因突变频率（Variant allele frequency, VAF）≥2％的基因突变纳入分析，所有检测出的外显子区通过千人基因组计划（1000 Genomes Project）、COSMIC（癌症中的体细胞突变目录）及PolyPhen-2（Polymorphism Phenotyping 2）数据库筛选出致病基因。具体方法详见文献[14]。

3. 研究队列分组标准：PD定义为骨髓原始细胞增加≥50％达到5％（初次测序时骨髓原始细胞<5％者）或增加≥50％达到10％～20％（初次测序时骨髓原始细胞5％～10％者），或有下列任何一项：①ANC或PLT较最佳缓解疗效时下降≥50％；②HGB下降≥20 g/L；③出现输血依赖[3]。LT定义为初次测序时骨髓或外周血原始细胞<20％者骨髓或外周血原始细胞≥20％。非PD/LT指不符合PD和LT的标准。对84例患者进行分组，51例纳入非PD/LT组，20例纳入PD组，13例纳入LT组。

4. 病程中基因突变动态变化分析指标：①新增突变（Ⅰ类突变）：初次测序时未检出，病程中新检出的基因突变；②克隆扩增突变（Ⅱ类突变）：初次测序时检出，病程中VAF增加≥10％的基因突变；③克隆缩小突变（Ⅲ类突变）：初次测序时检出，病程中VAF减少≥10％的基因突变或基因突变未再次检出；④克隆稳定突变（Ⅳ类突变）：初次测序时检出，病程中VAF增加或减少<10％的基因突变。

5. 治疗方案：2例（2.4％）患者仅接受输血支持治疗，16例（19.0％）接受免疫调节与促造血治疗，27例（32.1％）接受单用去甲基化药物治疗，34例（40.5％）去甲基化药物联合其他药物（维奈克拉、来那度胺、高三尖杉酯碱、阿糖胞苷）治疗，5例（6.0％）接受异基因造血干细胞移植治疗。

6. 疗效评价标准：MDS疗效判定依据《骨髓增生异常综合征中国诊断与治疗指南（2019年版）》[3]疗效标准，以患者两次测序期间治疗达到的最佳疗效来进行疗效分析，总有效率（Overall response rate，ORR）定义为完全缓解（Complete remission，CR）、部分缓解（Partial remission, PR）、骨髓完全缓解（Bone marrow complete remission, mCR）及血液学改善（Hematology improvement, HI）率之和。

7. 随访：随访截至2024年2月29日，随访资料来源于住院/门诊病历及电话随访记录。患者总生存（Overall survival, OS）时间按初次测序时间起至死亡或随访截止日期计算。

8. 统计学处理：应用SPSS 24.0软件进行分析。计量资料符合偏态分布，以中位数（范围）表示，组间比较采用Mann-Whitney *U*检验，率的比较采用卡方检验或Fisher确切概率法，生存分析采用Kaplan-Meier法。双侧*P*<0.05为差异有统计学意义。

## 结果

一、初次测序时临床特征

84例患者中男51例，女33例，初次测序时中位年龄69（31～95）岁；依据WHO2022诊断标准：MDS伴低原始细胞（MDS-LB）51例、MDS-5q− 1例，MDS伴SF3B1突变（MDS-SF3B1）7例、MDS伴TP53双等位突变（MDS-biTP53）7例、MDS伴骨髓纤维化（MDS-f）1例、MDS伴原始细胞增多Ⅰ型（MDS-IB1）9例、MDS伴原始细胞增多Ⅱ型（MDS-IB2）8例。中位HGB 73.5（42～136）g/L，中位WBC 2.54（0.75～37.53）×10^9^/L，中位PLT 59.5（9～1 605）×10^9^/L，中位ANC 1.42（0.01～34.35）×10^9^/L，中位外周血原始细胞比例0（0～9％），中位骨髓原始细胞比例0.4％（0～18.0％）。

70例染色体核型可分析的患者中，27例（38.6％）染色体核型异常，其中复杂染色体核型12例（17.15％）。IPSS-R细胞遗传学分组：染色体核型预后良好组46例（65.7％）、预后中等组12例（17.15％）、预后极差组12例（17.15％）。IPSS-R预后分组：极低危组4例（5.7％）、低危组18例（25.7％）、中危组28例（40.0％）、高危组9例（12.9％）、极高危组11例（15.7％）。比较PD/LT组与非PD/LT组患者初次测序时临床特征，结果见表1，PD/LT组骨髓原始细胞比例显著高于非PD/LT组（1.6％对0.4％，*P*＝0.013）。

**表1:** PD/LT组与非PD/LT组骨髓增生异常综合征患者临床特征比较

临床特征	PD/LT组（33例）	非PD/LT组（51例）	*P*值
年龄［岁，*M*（范围）］	69（40～89）	68（31～95）	0.721
男性［例（％）］	21（63.6）	30（58.8）	0.819
HGB［g/L，*M*（范围）］	75（42～136）	73（44～128）	0.637
WBC［×10^9^/L，*M*（范围）］	2.24（0.75～34.83）	3.17（0.81～37.53）	0.167
ANC［×10^9^/L，*M*（范围）］	1.11（0.15～32.49）	1.80（0.05～34.35）	0.080
PLT［×10^9^/L，*M*（范围）］	78（11～1605）	47（9～872）	0.088
外周血原始细胞比例［％，*M*（范围）］	0（0～9）	0（0～9）	0.848
骨髓原始细胞比例［％，*M*（范围）］	1.6（0～18.0）	0.4（0～11.2）	0.013
WHO2022分型［例（％）］			0.436
MDS-LB-SF3B1	4（12.1）	3（5.9）	
MDS-biTP53	4（12.1）	3（5.9）	
MDS-LB	16（48.5）	35（68.6）	
MDS-IB1	5（15.2）	4（7.8）	
MDS-IB2	4（12.1）	4（7.8）	
MDS-f	0（0）	1（2.0）	
MDS-5q−	0（0）	1（2.0）	
IPSS-R细胞遗传学分组［例（％）］			0.192
好	14（53.8）	32（72.7）	
中等	5（19.2）	7（15.9）	
极差	7（29.6）	5（11.4）	
IPSS-R预后分层［例（％）］			0.284
极低危	2（7.7）	2（4.5）	
低危	4（15.4）	14（31.8）	
中危	9（34.6）	19（43.2）	
高危	5（19.2）	4（9.1）	
极高危	6（23.1）	5（11.4）	

**注** PD：疾病进展；LT：白血病转化；MDS-LB-SF3B1：MDS伴SF3B1突变；MDS-biTP53：MDS伴TP53双等位突变；MDS-LB：MDS伴低原始细胞；MDS-IB1：MDS伴原始细胞增多Ⅰ型；MDS-IB2：MDS伴原始细胞增多Ⅱ型；MDS-f：MDS伴骨髓纤维化；MDS-5q−：MDS伴5号染色体长臂缺失；IPSS-R：修订版国际预后积分系统

二、初次测序时分子生物学特征

84例患者初次测序基因突变结果见图1A，初次测序时检出率较高的基因突变依次为ASXL1（21例，25.0％）、TP53（17例，20.2％）、TET2（12例，14.3％）、DNMT3A（11例，13.1％）、U2AF1（11例，13.1％）。PD/LT组中位基因突变个数显著高于非PD/LT组［2（0～9）个对1（0～7）个，*P*＝0.014］（图1B），其中TET2（27.3％对5.9％，*P*＝0.010）、SETBP1（15.2％对2.0％，*P*＝0.033）、RUNX1（18.2％对2.0％，*P*＝0.013）突变比例显著高于非PD/LT组（图1C）。

**图1:**
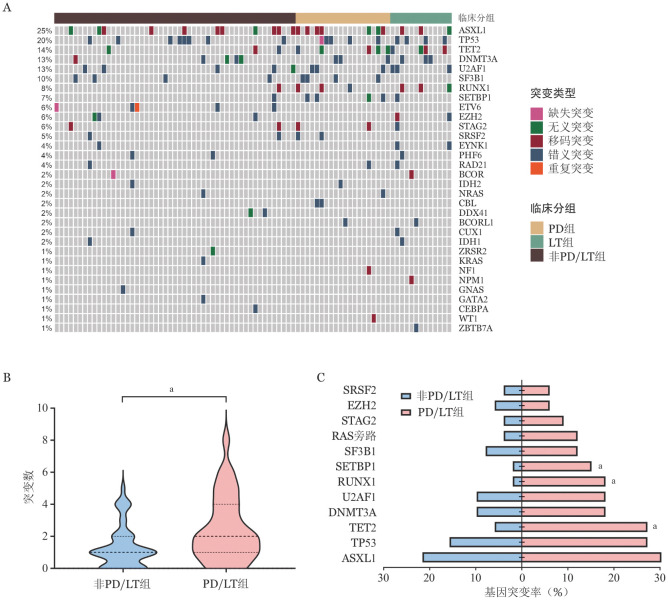
84例骨髓增生异常肿瘤患者初次测序基因突变情况 **A** 不同个体基因突变及突变类型的热图；**B** 疾病进展/白血病转化（PD/LT）组与非PD/LT组基因突变个数比较（^a^*P*<0.05）；**C** PD/LT组与非PD/LT组不同基因突变比例比较

三、病程中基因突变动态变化

84例患者两次测序中位间隔时间8.5（1～45）个月，PD/LT组和非PD/LT组患者两次测序中位间隔时间差异无统计学意义［7.5（1～45）个月10（1.5～41）个月，*P*＝0.355］。病程中基因突变动态变化结果见图2A，检出率较高的Ⅰ/Ⅱ类突变基因依次为TP53（9例，10.7％）、TET2（7例，8.3％）、ASXL1（7例，8.3％）、RAS旁路（包括NRAS、KRAS、PTPN11、CBL、NF1）突变（7例，8.3％）。PD/LT组Ⅰ/Ⅱ类突变数目显著高于非PD/LT组［2（0～6）个对0（0～3）个，*P*<0.001］（图2B）。PD/LT组患者Ⅰ/Ⅱ类TP53（27.3％对0，*P*<0.001）、RAS旁路（包含NRAS，KRAS，CBL，PTPN11，NF1基因）（21.2％对0，*P*＝0.001）、TET2（18.2％对2.0％，*P*＝0.013）突变比例显著高于非PD/LT组（图2C）。提示Ⅰ/Ⅱ类TP53、RAS旁路、TET2突变可能是促使MDS患者发生PD/LT的关键基因突变。

**图2:**
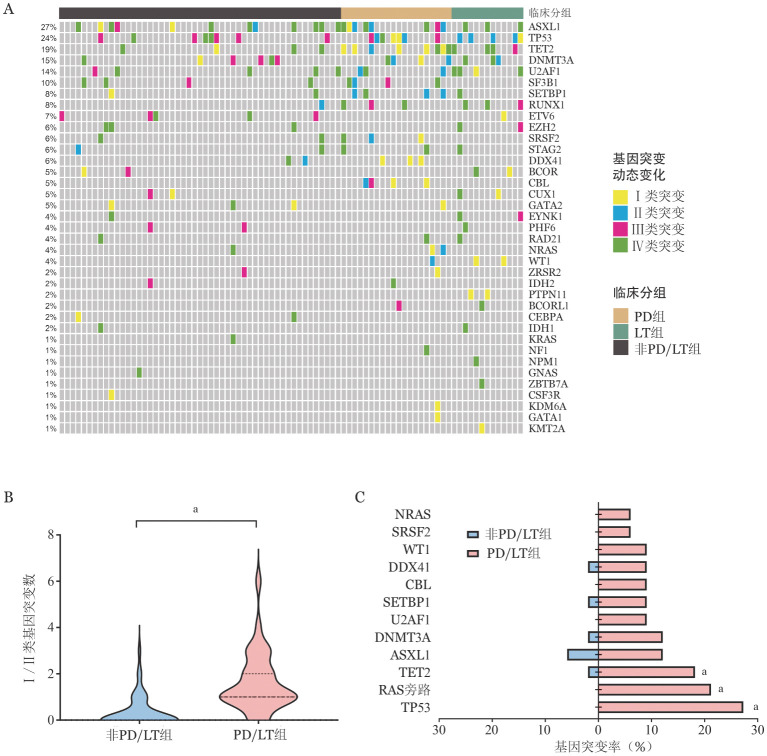
84例骨髓增生异常肿瘤患者病程中基因突变情况 **A** 不同个体基因突变及突变类型的热图；**B** 疾病进展/白血病转化（PD/LT）组与非PD/LT组Ⅰ/Ⅱ类基因突变个数比较（^a^*P*<0.05）；**C** PD/LT组与非PD/LT组不同基因Ⅰ/Ⅱ类突变比例比较

四、PD/LT过程中关键基因突变动态变化

分析MDS病程中TP53突变动态变化，结果示12例病程中有TP53突变的PD/LT组患者中9例（75.0％）为Ⅰ/Ⅱ类突变；而8例病程中有TP53突变的非PD/LT类患者5例为Ⅲ类、3例为Ⅳ类突变，无Ⅰ/Ⅱ类突变（图3A、3B）。

**图3:**
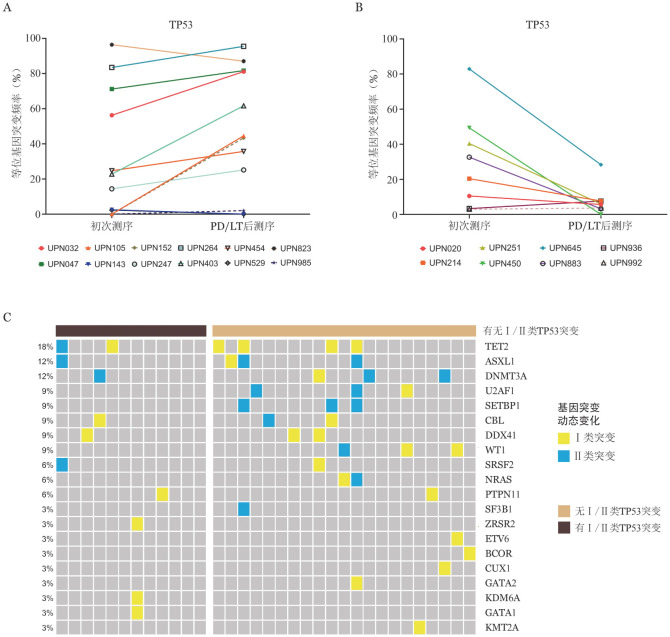
骨髓增生异常肿瘤患者病程中TP53突变动态变化图 **A** 疾病进展（PD）/白血病转化（LT）组病程中TP53突变动态变化；**B** 非PD/LT组病程中TP53突变动态变化；**C** PD/LT组病程中无Ⅰ/Ⅱ类TP53突变患者伴随的其他Ⅰ/Ⅱ类基因突变图

进一步分析PD/LT组中无Ⅰ/Ⅱ类TP53突变患者病程中伴随的其他Ⅰ/Ⅱ类基因突变，结果示最常见的Ⅰ/Ⅱ类基因突变依次为RAS旁路（5/24，20.8％）、TET2（5/24，20.8％）、ASXL1（4/24，16.6％）、SETBP1（5/24，16.6％）、DDX41（3/24，12.5％）、WT1（3/24，12.5％）突变，PD/LT组中有3例患者病程中TP53突变无克隆扩增，其中1例伴有Ⅰ类DDX41突变；1例伴有Ⅰ类ZRSR2、KDM6A、GATA1突变；1例伴有Ⅰ类SETBP1突变和Ⅱ类TET2、ASXL1、SRSF2突变。

分析MDS患者病程中RAS旁路突变动态变化，结果示8例病程中有RAS旁路突变的PD/LT组患者中7例为Ⅰ/Ⅱ类基因突变；而非PD/LT组患者仅有1例病程中有RAS旁路突变，为Ⅳ类突变。

分析MDS患者病程中TET2突变动态变化，结果示12例病程中有TET2突变的PD/LT组患者中，6例为Ⅰ/Ⅱ类突变；而4例病程中有TET2突变的非PD/LT组患者中，1例为Ⅰ类突变。

五、基因突变动态变化对患者预后及治疗效果影响分析

分析各基因动态变化对患者预后的影响，结果示病程中有Ⅰ/Ⅱ类TP53突变的患者OS期为7（95％*CI* 4.81～9.20）个月，病程中有Ⅲ/Ⅳ类TP53突变患者OS期为34.5（95％*CI* 8.29～60.71）个月；差异有统计学意义（*P*＝0.022），提示Ⅰ/Ⅱ类TP53突变是患者不良预后因素。

分析患者病程中基因突变动态变化对治疗疗效的影响，结果示病程中有Ⅰ/Ⅱ类基因突变患者的ORR显著低于病程中没有Ⅰ/Ⅱ类基因突变的患者（22％对48％，*P*＝0.010）。其中病程中有Ⅰ/Ⅱ类TP53突变的患者ORR低于有Ⅲ/Ⅳ类TP53突变的患者（0对36.4％），在0.1水平差异有统计学意义（*P*＝0.094）（表2）。

**表2:** 骨髓增生异常肿瘤患者病程中不同基因突变对治疗疗效影响［例（％）］

突变类型	治疗有效	治疗无效	*P*值
TP53突变			0.094
Ⅰ/Ⅱ类	0（0）	9（100.0）	
Ⅲ/Ⅳ类	4（36.4）	7（63.6）	
ASXL1突变			1.000
Ⅰ/Ⅱ类	2（28.6）	5（71.4）	
Ⅲ/Ⅳ类	4（25.0）	12（75.0）	
TET2突变			0.475
Ⅰ/Ⅱ类	0（0）	7（100.0）	
Ⅲ/Ⅳ类	2（28.6）	7（77.8）	
U2AF1突变			0.371
Ⅰ/Ⅱ类	0（0）	3（100.0）	
Ⅲ/Ⅳ类	2（22.2）	7（77.8）	
DNMT3A突变			0.576
Ⅰ/Ⅱ类	1（20.0）	4（80.0）	
Ⅲ/Ⅳ类	4（50.0）	4（50.0）	
RAS旁路突变			1.000
Ⅰ/Ⅱ类	0（0）	2（100.0）	
Ⅲ/Ⅳ类	0（0）	7（100.0）	
SETBP1突变			1.000
Ⅰ/Ⅱ类	0（0）	4（100.0）	
Ⅲ/Ⅳ类	0（0）	3（100.0）	

## 讨论

MDS是一种异质性较大的疾病，不同亚型的患者预后差异大[15]，发生PD/LT是严重影响患者长期生存的危险因素；如何阻止MDS患者发生PD/LT，维持MDS患者疾病稳定是临床研究的难题。为阐释MDS患者PD/LT中的基因突变动态变化，我们分析了本中心84例MDS患者序贯样本测序结果，以探究促使MDS患者发生PD/LT的关键基因突变，为未来开发针对这些基因突变及相关通路的小分子靶向抑制剂提供理论基础。

本研究中PD/LT组患者初次测序时中位骨髓原始细胞比例，中位基因突变个数，TET2、RUNX1、SETBP1突变比例显著高于非PD/LT组，提示这些临床与分子生物学特征是发生PD/LT的危险因素，与既往研究结果相似[4],[16]–[17]。本研究中PD/LT患者Ⅰ/Ⅱ类基因突变数目较非PD/LT组患者显著增加，与既往研究结果[11]–[12]一致，提示MDS疾病进展与新增恶性克隆和原有恶性克隆扩增相关；而Bernard等[17]研究了对MDS患者预后有显著影响的基因突变，并在此基础上提出了分子国际预后积分系统（Molecular international prognostic scoring system, IPSS-M），Wu等[18]则在中国60岁以上MDS患者中证实IPSS-M的预后评估价值要高于IPSS-R；因此推荐有条件的MDS患者应定期接受二代测序基因突变检测，这样既能尽早发现可能导致PD/LT的Ⅰ/Ⅱ类基因突变，也能根据IPSS-M预后分组及时调整治疗方案。

既往研究证实，TP53突变是MDS患者预后不良和发生LT的危险因素[19]–[23]，并发现TP53突变对OS和LT的影响与TP53突变类型和VAF大小相关。Bernard等[17]发现TP53双等位突变与不良预后和高LT风险相关，而TP53单等位突变OS和LT风险与无突变患者无明显差异；Montalban-Bravo等[23]发现，TP53突变VAF与不良预后和LT显著相关，TP53突变VAF≥10％的患者其OS显著差于TP53突变VAF<10％的患者，并发现在MDS患者LT过程中，TP53突变VAF有显著上升，与本研究结果相似。本研究中的新发现在于，初次测序时有TP53突变并不一定会使MDS患者发生PD/LT，只有病程中新增/克隆扩增的TP53突变才会促使MDS患者发生PD/LT，若治疗能使TP53突变VAF降低，则患者不易发生PD/LT；提示如果能降低TP53突变VAF，抑制TP53突变恶性克隆扩增，可能可以阻止MDS患者发生PD/LT，因此针对TP53突变的小分子靶向抑制剂可能是MDS患者治疗的新思路[24]。

本研究我们发现病程中Ⅰ/Ⅱ类RAS旁路突变可促使MDS患者发生PD/LT，与既往研究结果[11]–[12]一致，进一步证实了新增/克隆扩增RAS旁路突变是导致MDS患者发生PD/LT的关键基因，提示抑制RAS旁路突变克隆扩增可能可以阻止MDS患者发生PD/LT，因此未来可开展针对RAS旁路突变的小分子靶向抑制剂治疗伴有RAS旁路突变的MDS患者的相关临床试验。

Lin等[16]研究表明，有TET2突变的MDS患者无白血病生存期短于无TET2突变患者，并在序贯样本研究中发现MDS LT过程中存在原有TET2突变克隆扩增现象，但未检出新增TET2突变，且初次测序时有TET2突变患者LT过程中皆存在其他Ⅰ/Ⅱ类突变；本研究中PD/LT组患者病程中50％ TET2突变为Ⅰ/Ⅱ类突变，但这些Ⅰ/Ⅱ类TET2突变多数同时也伴有其他Ⅰ/Ⅱ类突变，与该项研究[16]结果相似，提示TET2突变可能促进了MDS患者发生PD/LT，但不是PD/LT的直接原因。

本研究我们发现病程中Ⅰ/Ⅱ类突变患者的治疗有效率显著低于Ⅲ/Ⅳ类突变患者，提示病程中新增/克隆扩增的基因突变可能是治疗疗效的不良预后因素；因此治疗过程中动态进行疾病驱动和预后关键基因监测，有利于发现可能导致治疗反应差和疾病进展的Ⅰ/Ⅱ类基因突变并及时调整治疗方案。

总之，本研究结果初步揭示PD/LT组初次测序时中位骨髓原始细胞比例和基因突变数目高于非PD/LT组，TET2、SETBP1、RUNX1突变比例高于非PD/LT组；PD/LT组Ⅰ/Ⅱ类基因突变数目和Ⅰ/Ⅱ类TP53、RAS旁路、TET2突变比例高于非PD/LT组患者；Ⅰ/Ⅱ类TP53和RAS旁路突变促进了MDS患者发生PD/LT。本研究存在以下不足：①作为单中心研究，样本量较少，不可避免存在偏移。②因本研究患者接受的同一药物的剂量或疗程不同，且用药依从性差异较大，因此未对患者的治疗方案对MDS患者病程中基因突变动态变化影响进行分析。本研究结论有待全国多中心、大样本量的患者群体研究进一步证实。
